# LMP1-augmented kappa intron enhancer activity contributes to upregulation expression of Ig kappa light chain via NF-kappaB and AP-1 pathways in nasopharyngeal carcinoma cells

**DOI:** 10.1186/1476-4598-8-92

**Published:** 2009-10-27

**Authors:** HaiDan Liu, Hui Zheng, Zhi Duan, DuoSha Hu, Ming Li, SuFang Liu, ZiJian Li, XiYun Deng, ZhenLian Wang, Min Tang, Ying Shi, Wei Yi, Ya Cao

**Affiliations:** 1 Cancer Research Institute, Xiangya School of Medicine, Central South University, Xiangya Road 110, Changsha, Hunan 410078, PR China; 2 Center of Clinical Gene Diagnosis and Therapy, The Second Xiangya Hospital of Central South University, Renmin Road 139, Changsha, Hunan 410011, PR China

## Abstract

**Background:**

Expression of kappa gene is under the control of distinct cis-regulatory elements, including the kappa intron enhancer (iE_κ_) and the kappa 3' enhancer (3'E_κ_). The active enhancers and expression of immunoglobulin is generally considered to be restricted to B lymphocytes. However, accumulating evidence indicated that epithelial cancer cells, including nasopharyngeal carcinoma (NPC) cell lines, express immunoglobulins. The mechanisms underlying the expression of Igs in nonlymphoid cells remain unknown. On the basis of our previous finding that expression of kappa light chain in NPC cells can be upregulated by EBV-encoded latent membrane protein 1(LMP1) through the activation of NF-κB and AP-1 signaling pathways, we thus use NPC cells as model to further explore the molecular mechanisms of nonlymphoid cells expressing Ig kappa.

**Results:**

In this study, luciferase reporter plasmid containing human wild-type iE_κ_, and its derivative plasmids containing mutant binding sites for transcription factor NF-κB or AP-1 were constructed. Luciferase reporter assays demonstrate iE_κ _is active in Igκ-expressing NPC cells and LMP1 expression can upregulate the activity of iE_κ _in NPC cells. Mutation of the NF-κB or AP-1 site within and downstream the iE_κ_, inhibition of the NF-κB and AP-1 pathways by their respective chemical inhibitor Bay11-7082 and SP600125 as well as stable or transient expression of dominant-negative mutant of IκBα (DNMIκBα) or of c-Jun (TAM67) indicate that both sites are functional and LMP1-enhanced iE_κ _activity is partly regulated by these two sites. Gel shift assays show that LMP1 promotes NF-κB subunits p52 and p65 as well as AP-1 family members c-Jun and c-Fos binding to the κNF-κB and the κAP-1 motifs *in vitro*, respectively. Both chemical inhibitors and dominant negative mutants targeting for NF-κB and AP-1 pathways can attenuate the LMP1-enhanced bindings. Co-IP assays using nuclear extracts from HNE2-LMP1 cells reveal that p52 and p65, c-Jun and c-Fos proteins interact with each other at endogenous levels. ChIP assays further demonstrate p52 and p65 binding to the κB motif as well as c-Jun and c-Fos binding to the AP-1 motif of Ig kappa gene *in vivo*.

**Conclusion:**

These results suggest that human iE_κ _is active in Igκ-expressing NPC cells and LMP1-stimulated NF-κB and AP-1 activation results in an augmenting activation of the iE_κ_. LMP1 promotes the interactions of heterodimeric NF-κB (p52/p65) and heterodimeric AP-1 (c-Jun/c-Fos) transcription factors with the human iE_κ _enhancer region are important for the upregulation of kappa light chain in LMP1-positive nasopharyngeal carcinoma cells.

## Background

While considerable evidence has shown that immunoglobulins (Igs) "unexpectly" expressed in malignant tumors of epithelial origin [1-10], much less is known about the molecular mechanisms of nonlymphoid cells expressing Igs. In our previous work, we have also demonstrated that nonlymphoid NPC cells express immunoglobulin kappa light chain. In addition, we have found that EBV-encoded latent membrane protein 1 (LMP1) can upregulate the expression of kappa light chain in NPC cells and both NF-κB and AP-1 signaling pathways are involved in LMP1-augmented kappa light chain expression [1]. These results promote us using of NPC cell lines as model to further explore the mechanisms underlying the expression of Ig kappa in nonlymphoid cells.

Expression of kappa light chain gene is under the control of distinct cis-regulatory elements, including the kappa intron enhancer (iE_κ_) and the kappa 3' enhancer (3'E_κ_) [11,12], which are located within the J_κ_-C_κ _region and downstream of C_κ _region, respectively. Both enhancers are inactive at the pro-B and pre-B cell stages and active at the Igκ-expressing mature B cell and plasma cell stages. The activity of these enhancers in other non-kappa-producing cell lineages, such as T-lymphoid cells, epithelial cells and NIH3T3 fibroblasts, is generally silent [11,13]. Base on these, it is generally believed that the activation of iE_κ _and 3'E_κ _is required for immunoglobulin kappa gene expression and is B cell lineage-restricted events [14,15]. An interesting feature of kappa gene transcription is its inducibility. Certain agents, such as cycloheximide (CYC), phorbol esters and bacterial product lipopolysaccharide (LPS) can induce the activation of kappa enhancers and result in kappa gene expression at the pre-B cell stage [16]. Nucleation of transcription factors PU.1, PIP, c-Fos and c-Jun on the kappa 3' enhancer core can cause a very dramatic induction in 3'E_κ _activity in NIH3T3 fibroblasts, a cell in which the enhancer is normally silent [13]. These findings reinforce the possibility of nonlymphoid cells expressing Ig kappa by certain unidentified mechanisms and suggest that other extracellular factors, such as gene products encoded by viruses, are also likely to induce kappa enhancers' activation, finally result in kappa gene transcription and expression.

One viral protein, latent membrane protein 1, is considered as a major oncogenic protein encoded by EBV for its transform and tumorigenic activities and is found to be able to transform cell lines and alter the phenotype of cells due to its oncogenic potential [17]. Biologically, LMP1 is an integral membrane protein with six transmembrane segments that facilitate self-aggregation in the plasma membrane and transduces ligand-independent signals, including NF-κB, c-Jun-NH_2_-terminal kinase (JNK), p38/MAPK, Ras/MEK/ERK/MAPK, PI3K/Akt and JAK/STAT [18-23]. The nuclear factor κB (NF-κB) and c-Jun N-terminal kinase (JNK) signaling pathways are the most important, since their activation results in the overexpression of most LMP1 target genes [24]. LMP1 can mimic CD40 signaling to induce B cell activation and differentiation in vivo. They share some molecules such as TRAF1, 2, 3, and 5 as signal transducers as well as some pathways such as NF-κB, JNK, p38/MAPK, PI3K/Akt and JAK/STAT pathways [25]. In normal B cells, an important mechanism of Ig production is CD40 ligation-triggered cellular signaling pathways [26]. In addition, it has been found that CD40 signaling can increase IgH 3' enhancer activity [27]. These studies, in combination with our previous finding that kappa light chain is significantly higher in LMP1-positive than in LMP1-negative NPC cells [1], we thus speculate that upregulation the expression of kappa light chain by LMP1 may be the result of LMP1-induced kappa enhancer's activation in NPC cells.

The function of enhancers is mediated by DNA binding proteins that recruit to the enhancer [12]. Multiple protein binding sites have been identified and characterized in each of the kappa enhancers. A κB binding site within the iE_κ _and the activity of iE_κ _is mainly dependent on the nuclear factor NF-κB binding to κB element [28,29]. Deletion or mutation of the κB site abolishes the activity of iE_κ_, suggesting it might as a crucial enhancer element [16,30]. In addition, the human kappa gene J_κ_-C_κ _region also contains a perfect consensus AP-1 site, which located ~320 bases downstream of the κB site. The κAP-l site in the context of the iE_κ _can positively regulate the iE_κ _activity and kappa expression in B cells, suggests that it plays a role in kappa gene regulation [31]. However, in Igκ-expressing nonlymphoid cells, whether these two binding sites play roles in functional activation of iE_κ _is still unknown.

Since kappa enhancers' activation is required for Ig kappa gene expression and their activations are generally considered as B cell lineage-restricted events, and since NF-κB and AP-1 binding sites exist within and downstream the iE_κ _enhancer, and on the basis of our previous findings that both NF-κB and AP-1 pathways are involved in LMP1-augmented Ig kappa expression in human NPC cells, we therefore focus on the iE_κ _enhancer and attempt to study further whether it is active in Igκ-expressing NPC cells and whether LMP1-upregulated kappa expression is correlated with the activation of iE_κ _via NF-κB and AP-1 pathways. In this study, luciferase reporter analysis demonstrate that the iE_κ _whose activation is required for immunoglobulin kappa gene expression indeed activates in Igκ-expressing NPC cells and stable or transient LMP1 expression can upregulate the activity of iE_κ _in NPC cells. Moreover, mutation analysis of κB or AP-1 binding site within or downstream the iE_κ_, inhibition of LMP1-mediated NF-κB and AP-1 signaling pathways by using specific chemical inhibitors and dominant inhibitory molecules indicate that both sites are functional and LMP1-enhanced iE_κ _activity is regulated, to some extent, through these two sites. Gel shift assays show that LMP1 promotes NF-κB subunits p52 and p65 as well as AP-1 family members c-Jun and c-Fos binding to the κNF-κB and the κAP-1 motifs *in vitro*, respectively. Both chemical inhibitors and dominant negative mutants targeting for NF-κB and AP-1 pathways can attenuate theLMP1-enhanced bindings. Co-IP assays using nuclear extracts from HNE2-LMP1 cells reveal that p52 and p65, c-Jun and c-Fos proteins interact with each other at endogenous levels. ChIP assays further demonstrate p52 and p65 binding to the κB motif as well as c-Jun and c-Fos binding to the AP-1 motif of Ig kappa gene *in vivo*. Based on the findings reported here, we conclude that the iE_κ _enhancer is active in NPC cells and is further activated by LMP1 via NF-κB and AP-1 pathways, which contributes to the upregulation of Ig kappa by LMP1 in NPC cells.

## Results

### Activation of the human immunoglobulin kappa intron enhancer in Igκ-expressing nasopharyngeal carcinoma cells

**Figure 1:**
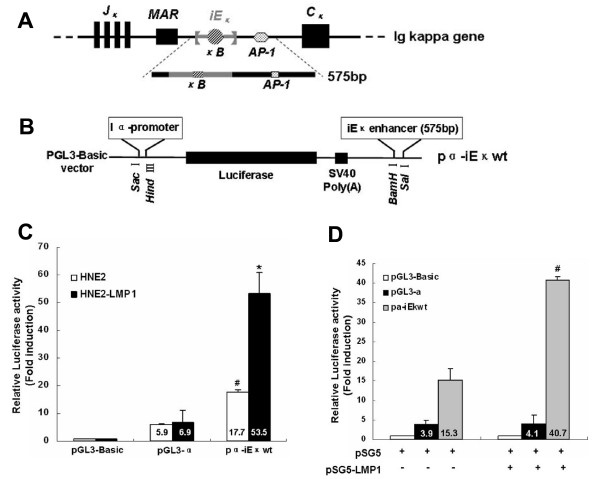
**Activation of the iE_κ _enhancer and enhancement of the iE_κ _activity by LMP1 in human nasopharyngeal carcinoma cells**. (A) Schematic diagram of human iE_κ_-containing DNA fragment used in these experiments. Position of the iE_κ_, the NF-κB and the AP-1 binding sites were shown. For simplicity, other protein-binding sites in the iE_κ _were not shown. (B) Insertion sites for the definite DNA fragment into the pGL3-α plasmid which contains the human immunoglobulin Iα promoter and the firefly luciferase reporter gene. (C) Comparison of the activities of iE_κ _in human nasopharyngeal carcinoma cell lines. Transient transfected the pα-iE_κ_wt construct, pGL3-α or pGL3-Basic vector into HNE2 and HNE2-LMP1 cells and luciferase reporter assays were performed as described in Materials and methods. The relative luciferase activity normalized to the value of the internal control plasmid pRL-SV40 activity. Results were expressed as fold induction of pGL3-Basic activity, which was assigned a value of 1. The data represent the mean ± SD of the three independent experiments performed in triplicate. Statistical significance: # P < 0.01 *vs. *pGL3-α-transfected HNE2, *P < 0.01 *vs. *pGL3-α-transfected HNE2-LMP1. (D) Transient expression of LMP1 increased the iE_κ _activity in nasopharyngeal carcinoma cells. HNE2 cells were co-transfected with 400 ng/well of pα-iE_κ_wt, pGL3-α or pGL3-Basic vector and 80 ng/well of internal control pRL-SV40 together with 200 ng/well of pSG5-LMP1 or blank expression plasmid pSG5 (total DNA ~800 ng). Cells were harvested at 36 h after transfection and subjected to luciferase analysis. Statistical significance: # P < 0.01 *vs. *pSG5-transfected HNE2.

Immunoglobulin kappa gene expression is under the control of distinct cis-regulatory elements, including the iE_κ _and the 3'E_κ _[11,12]. The activity of these enhancers is believed to contribute to Ig kappa expression in B cell lines [14]. In order to investigate if the iE_κ _enhancer could be functionally activated in NPC cells, we linked the iE_κ _to the Iα-promoter driving the transcription of the luciferase reporter gene and analyzed this reporter construct in transient transfection of NPC cell lines. A schematic diagram of the Ig kappa intron enhancer regions that were cloned was shown in Fig. 1A, and the wild-type reporter construct was illustrated in Fig. 1B. As shown in Fig. 1B, a 575 bp genomic fragment containing the intact iE_κ _was subcloned into the enhancerless pGL3-α plasmid. This construct, which containing wild-type κB site within the iE_κ _and wild-type AP-1 site downstream the 3' flank of the iE_κ _(designated as pα-iE_κ_wt), was introduced into HNE2 and HNE2-LMP1 cells to test the activity of iE_κ_. The human Iα promoter we used was identical to that used previously [32] and we found it to be minimally affected by LMP1 in our experiments (Fig. 1, C and 1D). Transfection of pα-iE_κ_wt generated higher luciferase activities than that of the pGL3-α construct whether in LMP1-negative (P < 0.01) or in LMP1-positive (P < 0.01) NPC cells (Fig. 1, C and 1D). Notably, the luciferase activity of pGL3-α in both HNE2 and HNE2-LMP1 cells was essentially equivalent, which suggested that the functional specificities of the iE_κ _enhancer in NPC cells were due to the enhancer itself rather than the promoter sequences. These results indicate that the iE_κ _is active in NPC cells which express immunoglobulin kappa light chain.

### Increases the activity of human immunoglobulin kappa intron enhancer by LMP1 in nasopharyngeal carcinoma cells

We found previously that EBV-LMP1 upregulates Ig kappa light chain expression in nasophayngeal epithelial cells [1]. In order to investigate whether the upregulation effect was due to LMP1-enhannced iE_κ _activity, luciferase reporter assays were performed to compare the iE_κ _activity in LMP1-positive and -negative NPC cells. The results indicated that the activity of iE_κ _in HNE2-LMP1 cells was significantly higher than that in HNE2 cells (Fig. 1C, 53.5 *vs. *17.7, P < 0.01), which was in line with the kappa chain expression patterns of these two cell lines [1]. Similar results were obtained with transient transfection of LMP1 into HNE2 cells (Fig. 1D). These results indicate that LMP1 can enhance the iE_κ _activity. We thus infer that LMP1 can increase the activity of iE_κ _and the upregulation of kappa light chain by LMP1 is likely due to increase the activity of iE_κ _by LMP1.

### Involvement of NF-κB and AP-1 signaling pathways in LMP1-increased human kappa intron enhancer activity

**Figure 2:**
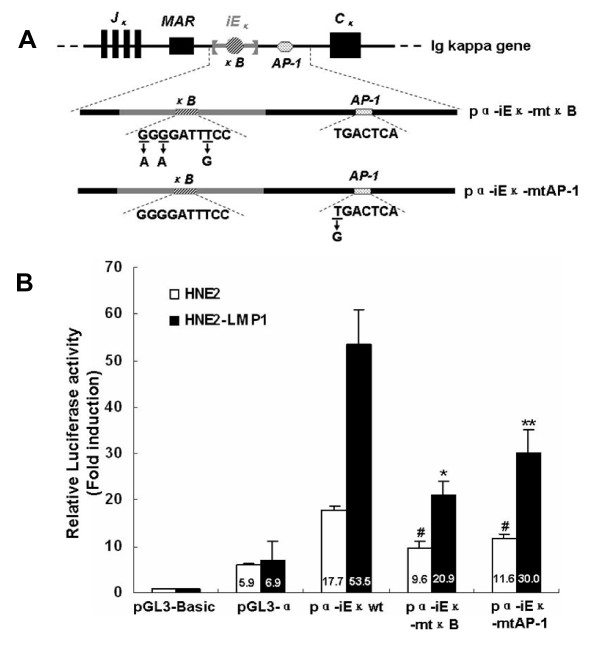
**Mutation of NF-κB or AP-1 binding site attenuated LMP1-increased iE_κ _activity**. (A) Schematic diagram of mutant iE_κ _constructs were shown. The expansion for NF-κB or AP-1 binding site gave its wild-type sequence and the nucleotides replaced by mutations were underlined. Arrows indicated nucleotides introduced by mutations. (B) Comparison of the activities of iE_κ _in human nasopharyngeal carcinoma cell lines. Transient transfected the constructs carrying wild-type NF-κB and AP-1 sequences (pα-iE_κ_wt), mutant NF-κB sequence (pα-iE_κ_-mtκB), mutant AP-1 sequence (pα-iE_κ_-mtAP-1), pGL3-α or pGL3-Basic into HNE2 and HNE2-LMP1 cells and luciferase reporter assays were performed as described in Materials and methods. The relative luciferase activity normalized to the value of the internal control plasmid pRL-SV40 activity. Results were expressed as fold induction of pGL3-Basic activity, which was assigned a value of 1. The data represent the mean ± SD of the three independent experiments performed in triplicate. Statistical significance: #P < 0.05 *vs. *pα-iE_κ_wt-transfected HNE2, * P < 0.01 and ** P < 0.05 *vs. *pα-iE_κ_wt-transfected HNE2-LMP1.

Based on the previous finding that both NF-κB and AP-1 signaling pathways are involved in LMP1-augmented kappa light chain expression [1], to determine whether either the NF-κB or the AP-1 sequence was required for LMP1-enhanced iE_κ _activity, site-directed mutagenesis by overlap extension PCR was used to introduce mutations into each of these sequences. From the pα-iE_κ_wt construct, we created constructs termed pα-iE_κ_-mtκB and pα-iE_κ_-mtAP-1, respectively (Fig. 2A). These constructs were introduced individually into human nasopharyngeal carcinoma cell lines to test the activity of iE_κ_. As shown in Fig. 2B, mutation of the NF-κB or the AP-1 motif significantly decreased LMP1-increased iE_κ _activity (p < 0.01 and p < 0.05, respectively). In addition, the magnitude of the reduction for pα-iE_κ_-mtAP-1 was less than that for pα-iE_κ_-mtκB, implying that of two signaling pathways, NF-κB pathway might play a leading role in LMP1-augmented iE_κ _activity in NPC cells. The activity of iE_κ _in HNE2 cells was moderately decreased by these genetic manipulations. Combination this with the results that mutation of either the NF-κB or the AP-1 motif could not completely abolish the iE_κ _activity in NPC cells as well as previous reports that several additional functional motifs are located within the iE_κ _[33,34], suggested that the variety of nuclear factors that can bind to the iE_κ _may result in complex regulatory pathways. Together, the results indicate that both NF-κB and AP-1 biding sites contribute to the basal and the LMP1-induced iE_κ _activities in NPC cells.

### Abrogation of LMP1-augmented human kappa intron enhancer activity by inhibitors and dominant negative mutants targeting for NF-κB and AP-1 pathways

**Figure 3:**
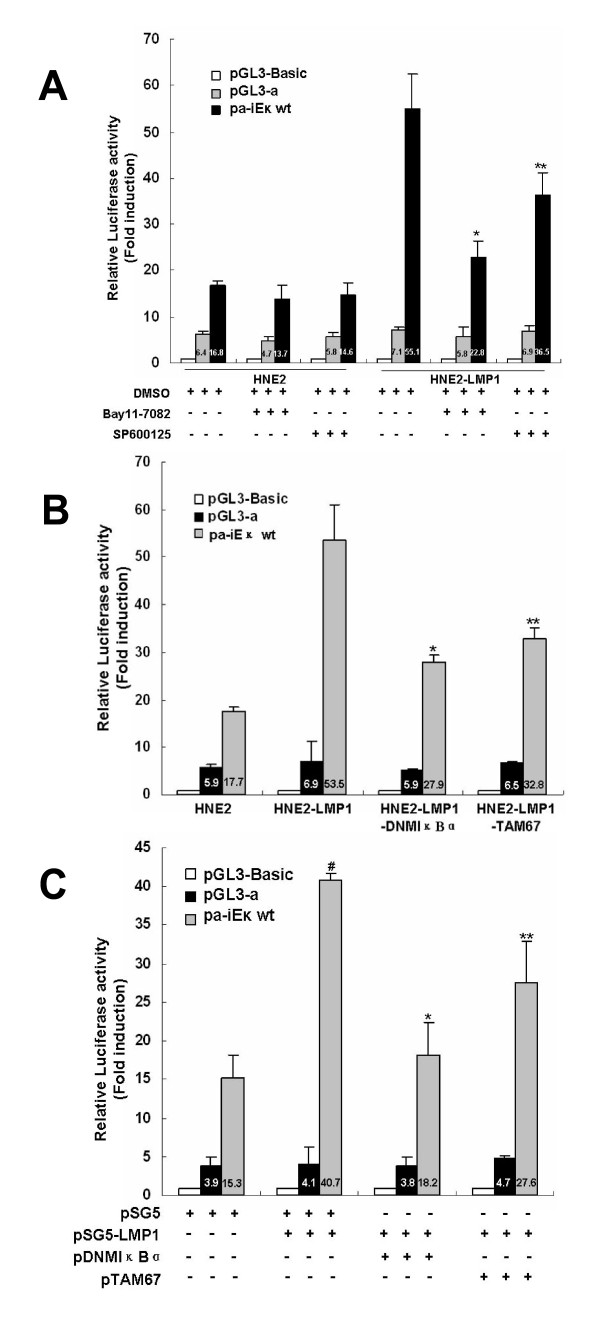
**Inhibitors and dominant negative mutants targeting for NF-κB and AP-1 pathways attenuated LMP1-augmented human iE_κ _activities**. (A) Bay11-7082 and SP600125 inhibited the activities of iE_κ _induced by stable expression LMP1. HNE2 and HNE2-LMP1 cells were transfected with pα-iE_κ_wt, pGL3-α or pGL3-Basic vector, and pRL-SV40 as an internal control for transfection efficiency. 24hr after transfection, cells were treated with Bay11-7082 (20 μM), SP600125 (20 μM) or 0.1% DMSO for 12hr. Cells were harvested at 36 h after transfection and subjected to the luciferase assay. Statistical significance: * P < 0.01 and ** P < 0.05 vs. HNE2-LMP1 vehicle control. (B) Stable expression DNMIκBα and TAM67 inhibited the activities of iE_κ _increased by LMP1. Indicated NPC cell lines were transfected with pα-iE_κ_wt, pGL3-α or pGL3-Basic vector, and pRL-SV40 as an internal control for transfection efficiency. Cells were harvested at 36 h after transfection and subjected to the luciferase assay. Statistical significance: * P < 0.01 and ** P < 0.05 vs. HNE2-LMP1 control. (C) Both DNMIκBα and TAM67 inhibited the activities of iE_κ _induced by transient expression LMP1. HNE2 cells were co-transfected with 400 ng/well of pα-iE_κ_wt, pGL3-α or pGL3-Basic vector and 80 ng/well of internal control pRL-SV40 together with 200 ng/well of pSG5-LMP1, pDNMIκBα or pTAM67 expression plasmid. The total amount of DNA (~800 ng) was kept constant by addition of blank expression plasmid pSG5 necessary to normalize the amount of DNA transfected. Cells were harvested at 36 h after transfection and subjected to the luciferase assay. Statistical significance: # P < 0.01 *vs. *pSG5-transfected HNE2, * P < 0.01 and ** P < 0.05 *vs. *pSG5-LMP1-transfected HNE2.

To further confirm both NF-κB and AP-1 sites contributed to LMP1-augmented iE_κ _activity, we used various specific inhibitors and dominant negative mutants for NF-κB and AP-1 signaling pathways to block the LMP1-mediated iE_κ _activation. As shown in Fig. 3A, LMP1-induced iE_κ _activity was significantly inhibited by 20 μM Bay11-7082 (p < 0.01) or 20 μM SP600125 (p < 0.05) but not by the DMSO vehicle control. These two compounds also decreased the iE_κ _activity in HNE2 cells to a certain extent but did not have statistical difference (P > 0.05), which was consistent with the previous immunoblot results that both compounds have no obvious inhibitory effects on kappa expression in HNE2 cells [1]. It was reported Bay11-7082 reduces only the constitutive but not the inducible activity of NF-κB [35]. We speculated SP600125 might reduce only the constitutive but not the inducible activity of JNK as did Bay11-7082, which might explain why both of them were not capable of decreasing the iE_κ _activity and kappa expression in HNE2 cells. Moreover, 20 μM Bay11-7082 showed more inhibitory effect on the activity of iE_κ _than 20 μM SP600125. We have found that the amount of kappa light chain in HNE2-LMP1-DNMIκBα and HNE2-LMP1-TAM67 cell lines is significantly lower than that in their parental cell line HNE2-LMP1 [1]. We therefore investigated whether the downregulation of kappa chain was correlated with the iE_κ _activity in the same cell lines. The results showed that the augmenting effect of iE_κ _activity by LMP1 was obviously attenuated when DNMIκBα and TAM67 were stably tranfected into HNE2-LMP1 cells (Fig. 3B). Transient co-transfection of DNMIκBα or TAM67 with LMP1 into HNE2 cells significantly declined the LMP1-upregulated iE_κ _activity (Fig. 3C). Together, these results again indicate that both NF-κB and AP-1 pathways play roles in the LMP1-upregulated iE_κ _activity in NPC cells.

### LMP1 promotes p52 and p65 binding to the κNF-κB motif as well as c-Jun and c-Fos binding to the κAP-1 motif in vitro

**Figure 4:**
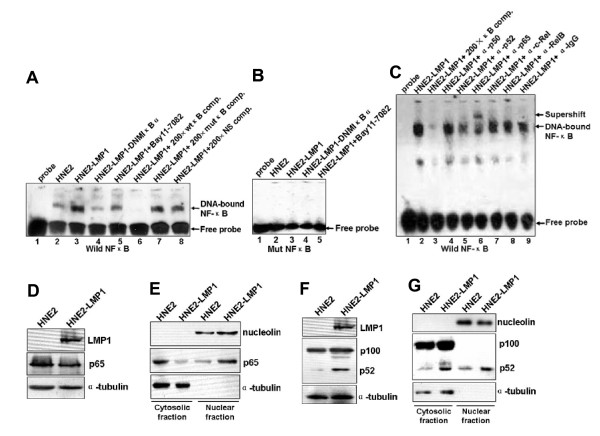
**LMP1 increased the binding ability of p52 and p65 transcription factors to κNF-κB motif *in vitro***. (A) Biotin-labeled wild-type κNF-κB oligonucleotide probe was incubated with nuclear extracts of HNE2, HNE2-LMP1, HNE2-LMP1-DNMIκBα and Bay11-7082-treated HNE2-LMP1 (20 μM for 12 hr) NPC cells in the presence of a 200-fold excess of unlabeled wild-type κNF-κB (lane 6), unlabeled mutant κNF-κB oligonucleotides (designated as mutκB, lane 7) or noncompetitive unlabeled κAP-1 oligonucleotide (NS, lane 8), and then NF-κB DNA binding activities were examined by EMSA. (B) Biotin-labeled mutant κNF-κB oligonucleotide probe was incubated with nuclear extracts of indicated NPC cell lines, and then NF-κB DNA binding activities were examined by EMSA. (C) 10 μg of HNE2-LMP1 nuclear extracts were preincubated with biotin-labeled κNF-κB oligonucleotide probe in the absence (lane 2) or presence of antibodies directed against different NF-κB subunits p50, p52, p65, c-Rel, RelB or control antibody (IgG) (indicated above each lane) and then supershift assays were performed. (D, E) Expression and subcelluar distribution of p65 in HNE2 and HNE2-LMP1 cells. Whole cell lysates, cytoplasmic and nuclear fractions were prepared and expression of p65 was estimated by western blotting. Cytosolic protein α-tubulin and nuclear protein nucleolin were detected as protein loading controls. (F, G) Expression and subcelluar distribution of p52 in HNE2 and HNE2-LMP1 cells. Whole cell lysates, cytoplasmic and nuclear fractions were prepared and expression of p52 was estimated by western blotting. Cytosolic protein α-tubulin and nuclear protein nucleolin were detected as protein loading controls.

**Figure 5:**
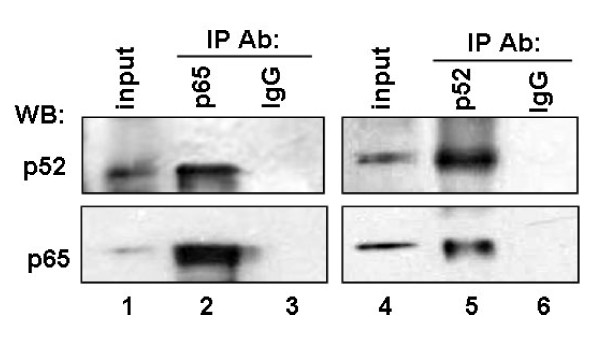
**Co-IP of endogenous p65 and p52**. Non-denatured nuclear extracts prepared from HNE2-LMP1 cells were immunoprecipitated with anti-p65, anti-p52 or control IgG antibody. The immune complexes and the input (10% of the nuclear extracts used in the immunoprecipitation step) were analyzed by immunoblotting with antibody specific to p52 (*upper panel*, lanes 1-3) or p65 (*lower panel*, lanes 4-6). The same membrane was stripped and reprobed to detect p65 (*lower panel*, lanes 1-3) or p52 (*upper panel*, lanes 4-6).

We demonstrated that the activity of iE_κ _was upregulated in HNE2-LMP1 cells and the activity of iE_κ _in the experimental NPC cell lines was consistent with their kappa chain expression patterns. To further investigate whether there was any correlation between our reporter expression and transcription factor binding activities of the DNA fragments covering the NF-κB and AP-1 motifs from the iE_κ_-containing J_κ_-C_κ _region of human kappa gene, we performed electrophoresis mobility shift assays to examine the protein complexes formed with κNF-κB and κAP-1 motifs at NPC cell lines. Biotin-labeled double-stranded κNF-κB and κAP-1 oligonucleotide probes as well as equal amounts of nuclear extracts from HNE2, HNE2-LMP1, HNE2-LMP1-DNMIκBα, HNE2-LMP1-TAM67, Bay11-7082-treated HNE2-LMP1 and SP600125-treated HNE2-LMP1 cells were used. As Fig. 4A shown, LMP1 caused a much stronger NF-κB DNA binding activity in HNE2-LMP1 cells than that in HNE2 cells (lane 2 *vs. *lane 3). The nuclear lysates isolated from HNE2-LMP1-DNMIκBα cells induced a weaker electromobility shift band than that from their parental cells HNE2-LMP1 (lane 3 *vs. *lane 4). We also found that the induction of NF-κB DNA binding activity by LMP1 was clearly inhibited by 20 μM Bay11-7082 (lane 3 *vs. *lane 5). To demonstrate the specificity of these interactions, competitive binding assays were performed. Excess unlabeled double-stranded κNF-κB oligonucleotide was included in the binding assay mixtures. A 200-fold excess of unlabeled oligonucleotide could completely compete for the protein binding seen with the HNE2-LMP1 cell extracts (lane 6). However, the same excess of the unlabeled mutant κNF-κB oligonucleotide (lane 7) or oligonucleotide containing the κAP-1 binding motif (lane 8) did not compete for the complex. In addition, the nuclear lysates isolated from these cell lines did not induce an electromobility shift when biotin-labeled κNF-κB mutant-type oligonucleotide was introduced (Fig. 4B). These implied that the complex formed with extracts was specific to the sequence of the κNF-κB oligonucleotide. To characterize the composition of the DNA-bound NF-κB complex, we performed super-EMSA with antibodies specific for NF-κB family members p50, p52, p65, c-Rel and RelB to analyze the nuclear extracts of HNE2-LMP1 cells. As shown in Fig. 4C, the addition of p50, c-Rel and RelB antibody did not influence the mobility or intensity of the NF-κB binding complex (lanes 4, 7 and 8), whereas the addition of antibodies for p52 and p65 resulted in a significant diminishment or supershift of the specific complex (lanes 5 and 6). A control IgG antibody failed to attenuate the shift or elicit a supershift (lane 9). The results indicate the presence of p52 and p65 proteins in the complex with the kappa NF-κB binding site. We further tested the effect of LMP1 on p65 and p52 expression. Though no obvious difference of p65 level in HNE2 and HNE2-LMP1 cells (Fig. 4D), by separating cytoplasmic and nuclear fractions, we found LMP1 led to p65 nuclear translocation (Fig. 4E). We also found LMP1 induced the processing of p100 to p52 (Fig. 4F) and the nuclear translocation of p52 (Fig. 4G). Efficient separation of the cytoplasmic and nuclear fractions was demonstrated by western blotting for cytoplasmic (α-tubulin) and nuclear (nucleolin) markers [36,37]. We next examined whether the interaction of p65 and p52 could be observed at endogenous levels. For this purpose, co-immunoprecipitation (co-IP) experiments were performed with non-denatured nuclear extracts from human nasopharyngeal carcinoma cell line HNE2-LMP1. As shown in Fig. 5, the p65 antibody could specifically coprecipitate endogenous p52 (Fig. 5, *upper panel*, compare lane 2 using p65 with lane 3 using control IgG antibody). Endogenous p65 could also be detected in a reverse co-IP experiment using p52 antibody in the IP step (Fig. 5, *lower panel*, lane 5). IgG was used as a negative control in the IP reaction. The protein input was shown as indicated. These results reveal a heterodimerization between p65 and p52, which is likely relevant to kappa light chain expression upregulated by LMP1 in NPC cells.

**Figure 6:**
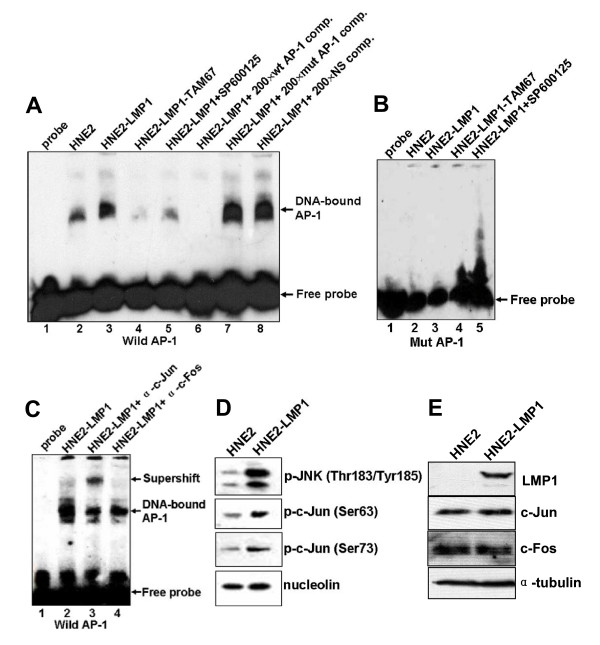
**LMP1 increased the binding ability of c-Jun and c-Fos transcription factors to κAP-1 motif *in vitro***. (A) Biotin-labeled wild-type κAP-1 oligonucleotide probe was incubated with nuclear extracts of HNE2, HNE2-LMP1, HNE2-LMP1-TAM67 and SP600125-treated HNE2-LMP1 (20 μM for 12 hr) NPC cells in the presence of a 200-fold excess of unlabeled wild-type κAP-1 (lane 6), unlabeled mutant κAP-1 oligonucleotides (designated as mutAP-1, lane 7) or noncompetitive unlabeled κNF-κB oligonucleotide (NS, lane 8), and then AP-1 DNA binding activities were examined by EMSA. (B) Biotin-labeled mutant κAP-1 oligonucleotide probe was incubated with nuclear extracts of indicated the NPC cell lines, and then AP-1 DNA binding activities were examined by EMSA. (C) 10 μg of HNE2-LMP1 nuclear extracts were preincubated with biotin-labeled κAP-1 oligonucleotide probe in the absence (lane 2) or presence of antisera directed against c-Jun (lane 3), c-Fos (lane 4) and then supershift assays were performed. (D) Phosphorylation levels of JNK and c-Jun in HNE2 and HNE2-LMP1 cells. The nuclear extracts were prepared and analyzed with phospho-JNK (Thr183/Tyr185), phospho-c-Jun (Ser63) and phospho-c-Jun (Ser73) antibodies, respectively. Nucleolin was acted as a nuclear protein loading control. (E) Expression of c-Jun and c-Fos in HNE2 and HNE2-LMP1 cells. Whole cell lysates were prepared and expression of c-Jun and c-Fos was estimated by Western blotting. α-tubulin was detected as a protein loading control.

**Figure 7:**
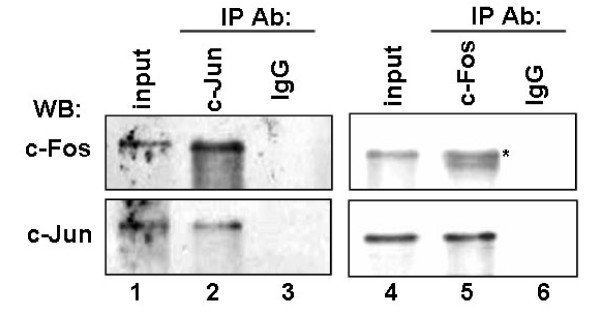
**Co-IP of endogenous c-Jun and c-Fos**. Non-denatured nuclear extracts prepared from HNE2-LMP1 cells were immunoprecipitated with anti-c-Jun, anti-c-Fos or control IgG antibody. The immune complexes and the input (10% of the nuclear extracts used in the immunoprecipitation step) were analyzed by immunoblotting with antibody specific to c-Fos (*upper panel*, lanes 1-3) or c-Jun (*lower panel*, lanes 4-6). The same membrane was stripped and reprobed to detect c-Jun (*lower panel*, lanes 1-3) or c-Fos (*upper panel*, lanes 4-6). Asterisks marked the specific bands recognized by c-Fos antibody.

Similarly, LMP1 increased the formation of AP-1 DNA binding complex (Fig. 6A, lane 2 *vs. *lane 3). The nuclear lysates isolated from HNE2-LMP1-TAM67 cells induced a weaker electromobility shift band than that from their parent cells HNE2-LMP1 (Fig. 6A, lane 3 *vs. *lane 4). The induction of AP-1 DNA binding activity by LMP1 was clearly inhibited by 20 μM SP600125 (Fig. 6A, lane 3 *vs. *lane 5). Protein binding to the κAP-1 probe was completely abrogated by a 200-fold excess of unlabeled wild-type κAP-1 probe (Fig. 6A, lane 6), but not by the same excess of unlabeled oligonucleotide probe containing mutation in the κAP-1 sequence (Fig. 6A, lane 7) and unlabeled κNF-κB probe (Fig. 6A, lane 8). On the other hand, the nuclear lysates isolated from these cells did not induce an electromobility shift when biotin-labeled κAP-1 mutant-type oligonucleotide was introduced (Fig. 6B). These implied that the complex formed with extracts was specific to the sequence of the κAP-1 oligonucleotide. To gain more insight into the composition of the protein complex bound to the human κAP-1 motif, we performed supershift analysis using nuclear extracts from HNE2-LMP1 cells. The addition of c-Jun antibody into the nuclear extracts of HNE2-LMP1 cells supershifted the complex (Fig. 6C, lane 3). Exposure of nuclear extracts from HNE2-LMP1 cells to c-Fos antibody and subsequent precipitation of the formed immune complex reduced the intensity of protein-DNA interaction by approximately 50% (Fig. 6C, lane 4). The super-EMSA results suggest that c-Jun and c-Fos are components of the complex bound to the human kappa AP-1 motif. Moreover, we found LMP1 could significantly upregulate JNK phosphorylation ([1] and Fig. 6D) and simultaneously upregulate the phosphorylation level of c-Jun at Ser63 and Ser73 in the nucleus (Fig. 6D). However, expression of c-Jun and c-Fos were essentially equal in HNE2 and HNE2-LMP1 cells ([1] and Fig. 6E). These results implied that LMP1-increased JNK activation led to the increased phosphorylation of c-Jun at Ser63 and Ser73, which might promote the JNK substrate c-Jun heterodimerize with c-Fos to form the AP-1 complex. To examine if c-Jun endogenously interacts with c-Fos, we performed co-IP experiments. As shown in Fig. 7, co-IP performed with anti-c-Jun antibody showed the co-precipitation with c-Fos from non-denatured nuclear extracts of HNE2-LMP1 cells (Fig. 7, *upper panel*, lane 2). Likewise, co-IP using anti-c-Fos antibody displayed c-Jun protein (Fig. 7, *lower panel*, lane 5). IgG was applied as a negative control in the IP reaction. The protein input was shown as indicated. These data show that the endogenous c-Jun and c-Fos associate *in vivo*.

Taken together, the results indicate that p52/p65 and c-Jun/c-Fos heterodimers can bind to the κB and the AP-1 site of human Ig kappa gene *in vitro*, respectively, which may be the key events in upregulating the activity of iE_κ _by LMP1 in NPC cells.

### LMP1 promotes p52/p65 binding to the κNF-κB motif as well as c-Jun/c-Fos binding to the κAP-1 motif in vivo

**Figure 8:**
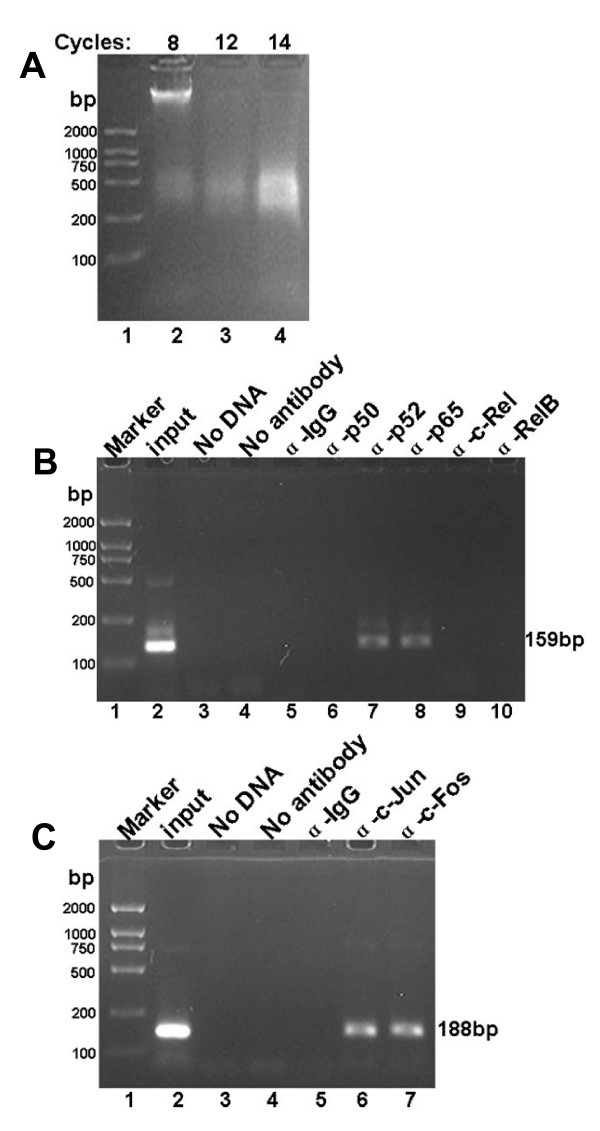
**p52/p65 and c-Jun/c-Fos heterodimers interacted with the human Ig kappa intronic enhancer and the adjacent sequence *in vivo***. (A) DNA sonication. 2% ethidium bromide-stained agarose gel demonstrated the shearing of chromatin from HNE2-LMP1 cells. Average chromatin length was ~500 bp. (B) ChIP analysis of the NF-κB binding site within the iE_κ _enhancer in HNE2-LMP1 cells. The cross-linked chromatin was precipitated with specific antibodies as indicated. The positive control was represented by the input fraction. Negative controls included a no chromatin sample, no antibody sample, and nonspecific antibody (αIgG). Precipitated DNA was analyzed by PCR using primers that amplified a 159-bp region that included the NF-κB site. (C) ChIP analysis of the AP-1 site located downstream of the iEκ enhancer. The PCR primers amplified a 188-bp sequence that included the AP-1 site.

To better understand p52/p65 and c-Jun/c-Fos heterodimers in the regulation of the human iE_κ _in vivo, we analyzed the fragments that span the NF-κB and the AP-1 binding regions within and downstream the iE_κ _using a chromatin immunoprecipitation assay (ChIP), respectively. The HNE2-LMP1 cells were treated with 1% formaldehyde to cross-link proteins to chromatin and the cross-linked chromatin was then sheared to fragments of ~500 bp in length via sonication (Fig. 8A). The sheared cross-linked chromatin was subsequently subjected to immunoprecipitation reactions using antibodies specific for the NF-κB family members p50, p52, p65, c-Rel and RelB as well as AP-1 family members c-Jun and c-Fos. An anti-IgG antibody was used as a nonspecific control. The precipitated chromatin DNA was then purified and amplified by PCR using primers specific for the NF-κB or the AP-1 binding site of Ig kappa gene. As shown in Fig. 8B, the primers for the human iE_κ _region containing the NF-κB binding site produced 159-bp amplicons that could be observed with the positive control (input chromatin) and when the chromatin was precipitated with antibodies specific for p52 and p65. Use of the p50, c-Rel, RelB antibody showed no positive signal and no amplification was observed with three negative controls (no chromatin, no antibody, and IgG). Another set of primers was used to analyze for *in vivo *AP-1 binding to the region located downstream the iE_κ _encompassing the AP-1 site. As shown in Fig. 8C, the AP-1 family members c-Jun and c-Fos antibodies could precipitate sequences that could be PCR-amplified and produced 188-bp amplicons using this second set of primers. Therefore, the ChIP results indicate that p52/p65 and c-Jun/c-Fos transcription factors can exert its regulatory function through direct binding to the human iE_κ _enhancer and the adjacent sequence.

## Discussion

In this article, we showed that the aberrant expression of Ig kappa light chain in NPC cells. Recent studies have demonstrated that the expression of Igs is widespread in epithelial cancers from many organs and includes basically all kinds of isotypes. Among heavy chains, α chain for IgA and γ chain for IgG are the mostly identified; but in light chain, only κ chain but not λ chain is confirmed. Moreover, several studies indicated that tumor-derived Igs have certain biological functions. Qiu et al [5] found induction of cancer cell apoptosis and inhibition of cancer growth by blocking tumor-derived IgG, whose light chain is kappa, using either antisense oligodeoxynucleotide or anti-human IgG, thus confirming that IgG secreted by epithelial cancers has some unidentified capacity to promote the growth and survival of tumor cells. We also found that blockade of cancer-derived Ig alpha suppresses the growth and viability of cancer cells. Furthermore, we have demonstrated that cancer-derived Ig alpha promotes the malignant proliferation ability of cancer cells and increases the access percentage of S phase from the early mitosis of synchronized cancer cells [38]. These findings support the important role of cancer-derived Ig as a growth factor of cancer cells. In addition, By *in situ *hybridization to analyze kappa constant region mRNA in different stages of cervical tissue samples, we found that the expression of kappa constant region mRNA is markedly increased in uterine cervical epithelia with dysplasia and carcinoma, as compared with cervicitis, thus suggesting a closely associated of kappa light chain expression with cell malignancy and is associated with increasing tumor grades [10]. Recently, we analyzed the ADCC immuno-activity of Ig derived from cancer cells and found that cancer-derived Ig is capable of reacting with FcR of monocytes and NK cells by its Fc region as does normal Ig, and to accomplish ADCC with effector cells (unpublished data). Based on these findings, it may be hypothesized that cancer-derived Ig could compete with B cell-derived Ig for the FcR on effector cells, thus inhibits ADCC and favors tumor immune escape. The potential biological functions of the tumor-derived Igs and the finding that nonlymphoid cells expressing Igs reported by different research groups revealed that this phenomenon is not a happenchance. However, the mechanisms underlying the expression of Igs in nonlymphoid cells are still unknown. In present study, we focus mainly on exploring the possible mechanisms by which nonlymphoid cells expressed Ig kappa and found that in Igκ-expressing NPC cells, kappa intron enhancer is activated. The activity of iE_κ _can be further activated by LMP1-stimulated NF-κB and AP-1 aberrant activation. It could be concluded that LMP1 stimulates transcription factors NF-κB and AP-1 binding to the corresponding site in kappa gene via NF-κB, JNK/MAPK signal pathways and finally upregulates kappa light chain induction. Such mechanisms would explain, at least in part, LMP1-positive human epithelial cancer cells produce immunoglobulins.

The activation of kappa enhancers, whose function is mediated by proteins binding to the enhancers, is required for Ig kappa gene expression [12,14,15]. We found the iE_κ _is active in both LMP1-negative and LMP1-positive NPC cells. In LMP1-negative HNE2 cells, the iE_κ _activity is relatively low and is in accord with low kappa expression level. LMP1 can further activate the activity of iE_κ _and contributes to the upregulation of Ig kappa in NPC cells. Our results indicated that mutant of either NF-κB or AP-1 biding site did not completely abolish the basal and LMP1-induced iE_κ _activities (Fig. 2B). In addition to NF-κB and AP-1 motifs to modulate the enhancer's activity, other positive regulatory elements have been identified within the iE_κ_, including κA and E-box motifs, these sequences could potentially regulate the activity of iE_κ _[33,34]. Therefore, other transcription factors bind to kappa gene through various signaling pathways to regulate kappa expression in NPC cells can not be excluded at this time.

In B cells, functional analyses of motifs within iE_κ _performed using isolated enhancers to activate reporter genes in transfection assays have shown that B cell-specific activity of iE_κ _depends substantially on the κB element [39]. Moreover, mutations of E-box motifs have variable and weaker effects on transcription compared to mutation of the κB site [16]. These observations indicated NF-κB acts as the master and commander of kappa gene expression via the κB motif in iE_κ _in B cells. Similarly, our result indicated mutation of the NF-κB motif displayed a more inhibitory effect on LMP1-increased iE_κ _activity compared to mutation of the AP-1 site, suggested that of NF-κB and AP-1 pathways, NF-κB pathway may play a leading role in LMP1-augmented iE_κ _activity in NPC cells.

Transcripition factor NF-κB comprised of homo- and heterodimers of the p65 (RelA), RelB, c-Rel, p50/p105 (NF-κB1) and p52/p100 (NF-κB2) polypeptides can both induce and repress gene expression by binding to discrete κB elements in promoters and enhancers [40]. NF-κB is found in the cytoplasm of pre-B cell lines as an inactive complexes associated with an IκB inhibitor, whereas in mature and transformed B cells, NF-κB is active and localized in the nucleus. NF-κB DNA binding activity and nuclear relocalization can be activated by a variety of stimuli. In our previous study, IκBα phosphorylation accompanying by IκBα degradation has been found in NPC cells and LMP1 can further induced IκBα phosphorylation and degradation [1]. Our results presented here indicated LMP1 increased the released-NF-κB translocating freely to the nucleus (Fig. 4E) and binding to the κB motif of iE_κ _(Fig. 4A, lane 3). We characterized the NF-κB/DNA complex containing p52 and p65 subunits by Gel Super-shift assay (Fig. 4C, lanes 5 and 6). We also found LMP1 induced the processing of p100 to p52 (Fig. 4F) and the nuclear translocation of p52 (Fig. 4G). Generally, p50/p65 is considered as a 'classical' heterodimers. p52 forms heterodimers with other NF-κB subunits, such as p65 and RelB, or as a homodimer has also been found [41]. However, in our experiments, we failed to detect p50, c-Rel and RelB subunits in NF-κB/DNA complex. We also confirmed the interaction of p52 and p65 at endogenous levels by co-IP assay (Fig. 5). Moreover, both p52 and p65 could directly bind to the NF-κB binding region within the iE_κ _enhancer (Fig. 8B). Perkins [42] found that p52/p65 preferentially activates HIV-1 gene expression relative to the p50/p65 heterodimers, which is similar to our results. The results suggest that a heterodimer of p65 with p52 subunit binding to κB site within the iE_κ _may play an important role in upregulating the activity of iE_κ _and kappa light chain production in HNE2-LMP1 NPC cells.

We reported earlier that NPC cells express activated forms of JNK (pJNK) whether LMP1-negative or LMP1-positive and LMP1 can increase the phosphorylation level of JNK [1]. JNK is one of the kinases that regulated the activation of AP-1 transcription factor. Upon stimulation, this protein kinase enters the nucleus to induce or phosphorylate subunits of AP-1 and the resultant enhanced AP-1 activity can then participate in the regulation of gene expression. The AP-1 transcription factor is a dimeric complex that comprises a group of structurally and functionally related members of the Jun family (c-Jun, JunB and JunD), Fos family (c-Fos, FosB, Fra-1 and Fra-2), ATF (ATFa, ATF-2 and ATF-3) and JDP (JDP-1 and JDP-2) subfamilies, which can bind to AP-1 consensus sequence 5'-TGAG/CTCA-3' [43]. Different types of AP-1 complexes are functionally distinct and may activate different target genes [44]. By EMSA analysis, we showed that nuclear extracts of both HNE2 and HNE2-LMP1 cells could bind κAP-1 motif and LMP1 was able to increase this binding (Fig. 6A, lanes 2 and 3). Super-EMSA further characterized the protein/DNA complex containing c-Jun and c-Fos transcription factors (Fig. 6C). Moreover, our results demonstrated LMP1-induced JNK phosphorylation level coincided with the phosphorylation level of c-Jun at Ser63 and Ser73 in the nucleus (Fig. 6D) and this c-Jun phosphorylation was much closely related to the DNA binding activity of the c-Jun/c-Fos heterodimer. Similar results that the phosphorylation level of c-Jun (ser63, ser73) is related to the DNA binding activity of c-Jun/JunB heterodimer was reported [23]. Our results suggest that LMP1 can act through activation of JNK, a c-Jun N-terminal kinase needed for AP-1 activation and induce formation of the c-Jun/c-Fos/DNA complex to upregulate the activity of iE_κ _in NPC cells. We also found stable expression of TAM67 almost completely blocked LMP1-induced AP-1 DNA binding in HNE2-LMP1 cells (Fig. 6A, lane 4). Similar results that stable TAM67 expression completely inhibited MKK6-induced AP-1 binding in MCF-7 cells [45] and an inhibition of nickel-induced AP-1 element binding by TAM67 in human bronchial epithelial cells [46] were recently reported. Although we have demonstrated the heterodimerization of c-Jun and c-Fos (Fig. 7) and this heterodimer can directly bind to the AP-1 site located near the iE_κ _enhancer (Fig. 8C), we have used only c-Jun and c-Fos in this report, therefore, other dimeric forms of AP-1 transcription factor involved in regulating the iE_κ _activity in NPC cells can not be excluded at this time.

## Conclusion

The present study presented novel experimental proofs on the mechanisms upregulating the expression of kappa light chain by LMP1 in NPC cells. Since other virus-encoded oncoproteins, such as HBX, E6, E7, can also activate many signal pathways including NF-κB and AP-1 pathways. These oncoproteins might induce immunoglobulin gene expression through the mechanism similar to EBV-LMP1. Our study might offer a new insight into the molecular mechanisms by which nonlymphoid cancer cells expressing immunoglobulin and lay foundations for further studies.

## Methods

### Cell lines and cell culture

HNE2, HNE2-LMP1, HNE2-LMP1-DNMIκBα and HNE2-LMP1-TAM67 cell lines used were as previously described [1]. All the cell lines were maintained in RPMI1640 (GIBCO) supplemented with 10% FBS (GIBCO), 1% glutamine, and 1% antibiotics at 37°C in humidified atmosphere with 5% CO2.

### Chemicals and cell treatments

The selective JNK inhibitor SP600125 (Cat No.420119, Calbiochem) and NF-κB inhibitor Bay11-7082 (Cat No.196870, Calbiochem) were prepared as a stock solution of 20 mM in dimethylsulfoxide (DMSO, Sigma). Subconfluent cells were treated with the compound at indicated concentrations for indicated time. Detailed treatment procedures were described in figure legends. The final concentration of DMSO in the culture media was kept less than 0.1% which had no significant effect on the cell growth.

### Plasmid constructs

The human Iα promoter was a 342 bp promoter fragment identical to that used previously [32], obtained by amplification from human HNE2 cells genomic DNA. The sense primer 5'-*gagctc*ctctgtctcggggtctctga-3' used in this reaction was carrying *Sac*I cloning site whereas the antisense primer 5'-*aagctt*ccgtctgtccttagcagagc-3' had *Hind *III site. Italic nucleotides represent restriction endonuclease recognition sites. This fragment was inserted into the *Sac *I/*Hind *III sites of the pGL3-Basic vector (Promega) and the plasmid was designated as pGL3-α.

A 575 bp fragment containing the intact human iE_κ _and the AP-l binding site at the 3' flank of iE_κ _was cloned. Briefly, a 575 bp DNA fragments containing human kappa light chain genomic sequences were amplified from HNE2 cells genomic DNA by PCR using specific primers from the human Ig kappa gene (GenBank accession no. NG_000834): 5'-*ggatcc*ctgacttctccctatctgtt-3'(sense), which contains an artificial *BamH *I site, and 5'-*gtcgac*ccattctgagggctttgc-3'(antisense), which contains an artificial *Sal *I site. Italic nucleotides represent restriction endonuclease recognition sites. The PCR-amplified fragments were then digested with *BamH *I/*Sal *I and inserted into the corresponding restriction sites of the pGL3-α plasmid described above to generate pα-iE_κ_wt. The PCR products were confirmed by their size, as determined by electrophoresis and by DNA sequencing. The NF-κB motif and the AP-1 motif mutants (designated as pα-iE_κ_-mtκB and pα-iE_κ_-mtAP-1, respectively) from pα-iE_κ_wt were generated by PCR based on an overlap extension technique [47]. The primers used for generating mutations were: 5'-ccccagag***a*g*a*gatt*g*cc**aagaggccacctg-3' and 5'-tt**gg*c*aatc*t*c*t***ctctgggggattc-3' (for NF-κB site), 5'-gaggctttcct***g*gactca**gccgctgcc-3' and 5'-gc**tgagtc*c***aggaaagcctccg-3' (for AP-1 site). PCR-amplified fragments carrying the desired mutations were then cloned into *BamH *I/*Sal *I sites of the pGL3-α plasmid. Bold nucleotides represent sequences of κNF-κB (**ggggatttcc**) and κAP-1(**tgactca**) motifs and bold italic nucleotides represent mutated nucleotides. The expected mutations and the integrity of the enhancer were confirmed by automated sequencing using an Applied Biosystems sequencer and software (Foster City, CA).

The pSG5-based expression vector for wild-type LMP1 derived from B95.8 EBV strain was kindly provided by Dr. Izumi (Brigham and Women's Hospital). Expression plasmid of dominant negative mutant of IκBα (DNMIκBα), which had a deletion of 71 amino acids at the N terminus and was cloned into the multiple cloning sites of pcDNA3, was kindly provided by Dr. Krappmann (Max-Delbruck-center for Molecular Medicine, Berlin, Germany). Expression plasmid of mutant c-Jun (TAM67) was constructed by inserting the TAM67 sequence into the vector pGem3z which contains a human keratin 14 promoter and a human growth hormone segment, was kindly provided by Dr. J. Li (NCI, Frederich, U.S.A.).

### Luciferase reporter assays

The pGL3-α, pα-iE_κ_wt, pα-iE_κ_-mtκB and pα-iE_κ_-mtAP-1 luciferase reporter plasmids described above were used in conjunction with the control pGL3-Basic vector (Promega) and the internal control plasmid pRL-SV40 (Promega). Cells were cultured in 24-well plates at a density of 1 × 10^5 ^per well overnight and were transfected with Lipofectamine™ 2000 (Invitrogen) as per the manufacturer's instructions. Each transfection contained 800 ng/well of firefly luciferase reporter and 80 ng/well of internal control pRL-SV40 or contained 400 ng/well of firefly luciferase reporter and 80 ng/well of internal control pRL-SV40 together with 200 ng/well of each expression plasmid or blank expression plasmid necessary to normalize the amount of DNA transfected. 24 hrs after transfection, cells were either left untreated or treated with 20 μM Bay11-7082, 20 μM SP600125 or 0.1% DMSO for 12 hrs. Cells were harvested at 36 h after transfection and lysates were analyzed for luciferase activity using the Dual Luciferase Reporter assay (Promega) according to the manufacturer's directions with a GloMax™ Microplate Luminometer (Promega). The luciferase reporter plasmids were co-transfected with pRL-SV40 to correct for variations in transfection efficiency. The relative luciferase activity normalized to the value of pRL-SV40 activity. Results were expressed as fold induction of pGL3-Basic activity, which was assigned a value of 1. The data represent the mean ± SD of the three independent experiments performed in triplicate.

### Western blot analysis

Whole cell lysates preparation and western blot analysis were performed according to the method previously described [1]. Nuclear or cytoplasmic extracts were prepared by the use of NE-PER Nuclear and Cytoplasmic Extraction Kit (Cat. No.78833, Pierce) in accordance with the manufacturer's protocol. Protein concentration was determined by BCA Assay Reagent (Cat. No.23228, Pierce). The following antibodies were used for immunodetection with appropriate dilutions: mouse LMP1 monoclonal antibody (CS.1-4, DAKO); p52(sc-298), p65(sc-8008), c-Jun(sc-44), c-Fos(sc-52), nucleolin(sc-8031), α-tubulin(sc-5286), goat anti-rabbit IgG-HRP (sc-2004), goat anti-mouse IgG-HRP (sc-2005) and donkey anti-goat IgG-HRP (sc-2020) (all from Santa Cruz); phospho-JNK(Thr183/Tyr185)(9251), phospho-c-Jun(Ser63)(9261S) and phospho-c-Jun (Ser73) (9164S) (all from Cell Signaling Technology).

### Electrophoretic mobility shift assay (EMSA)

Nuclear extracts were prepared by the use of NE-PER Nuclear and Cytoplasmic Extraction Kit (Cat. No.78833, Pierce) in accordance with the manufacturer's protocol. The protein concentration in nuclear extracts was determined using the BCA protein assay reagent (Cat. No.23228, Pierce) and EMSAs were carried out using aliquots containing equal amounts of protein. EMSA analysis was performed using the LightShift™ Chemiluminescent EMSA Kit (Cat. No.20148, Pierce) following the manufacturer's instructions. The reaction mixtures (20 μl) containing about 10 μg nuclear extracts were incubated with 2 nmol/L of the biotin-labeled double-stranded oligonucleotide probes in reaction buffer (Pierce) for 20 min at room temperature. Samples were subjected to electrophoresis in 5% nondenaturing polyacrylamide gel and transferred to Biodyne™ B Nylon membrane (Cat. No.77016, Pierce). For competition analyses, 200-fold excess of the unlabeled wild-type or mutant or nonspecific probe was included in the binding reaction. For antibody supershift experiments, the reaction mixtures were preincubated with 2 μg of p50(sc-8414X), p52(sc-298X), p65(sc-8008X), c-Rel(sc-272X), RelB(sc-226X), c-Jun(sc-44X), c-Fos(sc-52X) and rabbit IgG(sc-2027) antibody (all from Santa Cruz) at room temperature for 1 hr. The complementary oligonucleotides used as probes or competitors were listed below: the human κNF-κB oligonucleotides used were 5'-ccagag**ggggatttcc**aagaggcca-3' and 5'-tggcctctt**ggaaatcccc**ctctgg-3', derived from the sequence of the NF-κB site within the human kappa intron enhancer. The human κAP-l oligonucleotides used were 5'-gctttcct**tgactca**gccgctgcc-3' and 5'-ggcagcggc**tgagtca**aggaaagc-3', derived from the AP-1 sequence ~320 bp 3' of the κNF-κB site. The nonspecific oligonucleotides used as competitor DNA for κNF-κB and κAP-l were κAP-l and κNF-κB oligonucleotides, respectively. The mutated κNF-κB oligonucleotides used were 5'-ccagag***a*g*a*gatt*g*cc**aagaggcca-3' and 5'-tggcctctt**gg*c*aatc*t*c*t***ctctgg-3' (designated as mutκB). The mutated κAP-l oligonucleotides used were 5'-gctttcct***g*gactca**gccgctgcc-3' and 5'-ggcagcggc**tgagtc*c***aggaaagc-3' (designated as mutAP-l). Binding sites were shown in bold type and mutated nucleotides were shown in bold italic. The mutated oligo probes for NF-κB and AP-1 binding sites in EMSAs were identical to those of the mutated sequences in the reporter gene constructs.

### Co-immunoprecipitation (Co-IP)

Non-denatured nuclear proteins were purified using NE-PER Nuclear and Cytoplasmic Extraction Kit (Cat. No.78833, Pierce) according to the manufacturer's instructions. Protein concentration was determined by BCA Assay Reagent (Cat. No.23228, Pierce). 200 μg of nuclear extracts prepared from HNE2-LMP1 cells were mixed with 40 μl protein A-Sepharose beads (Sigma) in the immunoprecipitation assay buffer (1× PBS, 0.5% Nonidet P-40, 0.5% sodium deoxycholate, 0.1% SDS), incubated at 4°C for 2 h with gentle agitation and centrifuged for 2 min at 2000 rpm for preclearing. The recovered supernatant was incubated with 2 μg of an antibody to a member of the complex in the presence of 1× protease inhibitors at 4°C overnight with mild shaking. Then, 50 μl of protein A-Sepharose beads was added, and the incubation was continued for 2 h at 4°C with gentle shaking. Protein A-precipitated protein complex was recovered by brief centrifugation, followed by three times washes with immunoprecipitation assay buffer. The harvested beads resuspended in 30 μl of 2× SDS PAGE sample buffer were boiled for 5 min to release the bound protein. The samples were then analyzed by Western blot with a specific antibody to another member of the complex. A 20 μg aliquot of nuclear extract was used as an input control. The same membrane was stripped by incubating at 50°C for half an hour in stripping buffer [100 mM β-mercaptoethanol, 2% (wt/vol) sodium dodecyl sulfate and 62.5 mM Tris-HCl (pH 6.8)] and reprobed with the corresponding IP antibody.

### Chromatin immunoprecipitation (ChIP) assay

ChIP was performed using the ChIP assay kit (Upstate Biotechnology, Lake Placid, NY) and was then conducted according to the manufacturer's recommendations. Briefly, formaldehyde solution was added directly to HNE2-LMP1 cells at a final concentration of 1% at room temperature for 10 min. Then the cells was neutralized with glycine at room temperature for 5 min and washed twice with ice-cold 1× phosphate-buffered saline containing protease inhibitors. The cells were lysed by SDS lysis buffer with protease inhibitors. Chromatin in the lysate (350 μl) was sheared by sonication with a Branson Sonifier Cell disruptor B15 (output control 4, duty cycle 40%), with 14 cycles of 20-second pulses and 20-second intervals to an average length of ~500 bp as determined by 2% agarose gel electrophoresis. The suspension was precleared with salmon sperm DNA/protein A/agarose-50% slurry for 1 h at 4°C. After "precleared" the chromatin, a small aliquot (10 μl) was saved as "input DNA" for PCR analysis later. Other each 100 μl aliquots of sheared cross-linked chromatin were incubated with 2 μg each of antibodies p50(sc-8414X), p52(sc-298X), p65(sc-8008X), c-Rel(sc-272X), RelB(sc-226X), c-Jun(sc-44X), c-Fos(sc-52X), rabbit IgG(sc-2027) (Santa Cruz), or no Ab overnight at 4°C with mild shaking. The immune complexes were incubated with salmon sperm DNA/protein A/agarose-50% slurry with mild shaking for 2 h at 4°C, washed, and eluted. Cross-links were reversed by 5 M NaCl. After proteinase K digestion, DNA in samples was phenol extracted, ethanol precipitated, and resuspended in 50 μl of ddH_2_O. Two microliters of DNA solution was used for 36 cycles of PCR amplification. PCR products were analyzed by electrophoresis on a 2% agarose gel and visualized by ethidium bromide staining. The following primers were used in the ChIP assays: human iE_κ _enhancer including the NF-κB-binding region, 5'-ctactgctctcccacccaac-3' and 5'-tgcagcaattttcagccata-3'(159 bp); the AP-1-binding region located downstream the human iE_κ _enhancer, 5'-gcctgttatcccagcacagt-3' and 5'-tgcatgcttttctgaccttg-3'(188 bp).

### Statistical analysis

All statistical calculations were performed with the statistical software program SPSS ver.12.0. Differences between various groups were evaluated by the Student's *t *test. The difference was of statistical significance, when P < 0.05.

## Abbreviations

EBV: Epstein-Barr virus; NPC: nasopharyngeal carcinoma; LMP1: latent membrane protein 1; NF-κB: Nuclear factor kappa B; AP-1: activating protein-1; JNK: c-Jun N-terminal kinase; Ig: immunoglobulin; C_κ _region: kappa gene constant region; J_κ _region: kappa gene joining region; iE_κ_: immunoglobulin kappa intron enhancer; 3'E_κ_: immunoglobulin kappa 3' enhancer; EMSA: electrophoretic mobility shift assays; Co-IP: co-immunoprecipitation; ChIP: chromatin immunoprecipitation; TRAF: tumor necrosis factor receptor associated factor.

## Competing interests

The authors declare that they have no competing interests.

## Authors' contributions

HDL carried out all experiments and drafted the manuscript. HZ, ZD, DSH participated in the design of the study and assisted with the reporter gene analysis. SFL, ZJL participated in the statistical analysis. ML, XYD contributed in the design of the study and data interpretation. ZLW, MT, YS, WY assisted with the construction of luciferase reporter plasmids and participated in the sequence alignment. YC conceived of the study and participated in the design and coordination. All authors read and approved the final manuscript.
